# Effect of the Environmental Pollutant Hexachlorobenzene (HCB) on the Neuronal Differentiation of Mouse Embryonic Stem Cells ^†^

**DOI:** 10.3390/ijerph10105244

**Published:** 2013-10-21

**Authors:** Cynthia Addae, Henrique Cheng, Eduardo Martinez-Ceballos

**Affiliations:** 1 Department of Biological Sciences and Environmental Toxicology Program, Southern University and A&M College, Baton Rouge, LA 70813, USA; E-Mail: cynaddae@yahoo.com; 2 Department of Comparative Biomedical Sciences, School of Veterinary Medicine, Louisiana State University, Baton Rouge, LA 70803, USA; E-Mail: hcheng@vetmed.lsu.edu

**Keywords:** hexachlorobenzene, retinoid acid, reactive oxygen species, antioxidant, environmental pollutant, GABAergic, encapsulation, embryonic stem cells, autism, ADD

## Abstract

Exposure to persistent environmental pollutants may constitute an important factor on the onset of a number of neurological disorders such as autism, Parkinson’s disease, and Attention Deficit Disorder (ADD), which have also been linked to reduced GABAergic neuronal function. GABAergic neurons produce γ-aminobutyric acid (GABA), which is the main inhibitory neurotransmitter in the brain. However, the lack of appropriate models has hindered the study of suspected environmental pollutants on GABAergic function. In this work, we have examined the effect of hexachlorobenzene (HCB), a persistent and bioaccumulative environmental pollutant, on the function and morphology of GABAergic neurons generated *in vitro* from mouse embryonic stem (ES) cells. We observed that: (1) treatment with 0.5 nM HCB did not affect cell viability, but affected the neuronal differentiation of ES cells; (2) HCB induced the production of reactive oxygen species (ROS); and (3) HCB repressed neurite outgrowth in GABAergic neurons, but this effect was reversed by the ROS scavenger N-acetylcysteine (NAC). Our study also revealed that HCB did not significantly interfere with the function of K^+^ ion channels in the neuronal soma, which indicates that this pollutant does not affect the maturation of the GABAergic neuronal soma. Our results suggest a mechanism by which environmental pollutants interfere with normal GABAergic neuronal function and may promote the onset of a number of neurological disorders such as autism and ADD.

## 1. Introduction

Embryonic stem (ES) cells are derived from the inner cell mass (ICM) of blastocyte stage (day 3.5) embryos [1]. Due to their intrinsic properties, ES cells can be differentiated in culture into specific cell lineages with potential applications for the treatment of diseases such as spinal cord injuries, diabetes, stroke, heart disease, muscle damage, bone disorders, Parkinson’s and Alzheimer’s disease [2,3]. In this regard, it is possible that environmental pollutants such as hexachlorobenzene (HCB) may affect the differentiation of ES cells both *in vivo* and *in vitro*. HCB is a lipophilic and persistent pesticide that has been used to treat seeds of sorghum, wheat and other grains against fungi [4]. Although concerns about the adverse effects on the environment and human health resulted in the discontinuation of the use of HCB as a pesticide in many countries during the 1970s [5], human chronic exposure to low HCB doses is still a concern due to the persistent nature of this organochlorine pollutant [6].

In animals, HCB has been found to induce neurological symptoms such as paralysis, tremors, weakness, convulsions and muscle discoordination [7]. In humans, HCB has been shown to cause damage to the liver, thyroid, nervous system, bones, kidneys, blood, and immune and endocrine systems [4,8]. Furthermore, recent research suggests that exposure to low levels of HCB and other organochlorine compounds can affect young children’s neurological and behavioral development [9,10].

At the cellular level, HCB acts as a dioxin-like agent and binds to aryl hydrocarbon receptors (AhRs) [11]. This binding results in the modulation of gene expression and induction of important cellular processes [11,12,13]. For instance, Salmon *et al.* [14] exposed mouse and human embryonic cells to HCB and found that this compound induced cell membrane damage, a short-term decrease in cell number, increased DNA strand breaks, and a long-term decrease in colony survival.

Although many studies have been carried out to test the toxicity of HCB in mammalian cells, the effect of this pollutant on the neuronal differentiation of ES cells has not yet been examined. In this work, we employed a cell encapsulation protocol to generate GABAergic neurons from mouse ES cells and used it to examine the sub-toxic effect of HCB on neuronal differentiation. We first showed that the lowest dose of HCB tested (0.5 nM) did not significantly affect cell viability, but interfered with neurite outgrowth in GABAergic neurons generated from mouse ES cells. This effect was shown to result from the HCB-induced generation of Reactive Oxygen Species (ROS) and it was reversed by pretreatment with the ROS scavenger N-acetylcysteine (NAC). Interestingly, HCB treatment did not affect the function of voltage-gated K^+^ channels in the differentiated GABAergic neurons. Results from our experiments may have implications on the assessment of HCB neurotoxicity after exposure to low levels of this compound during pregnancy. Our results also suggest that maternal consumption of antioxidants may help prevent the onset of neurological diseases in fetuses that result from chronic exposure to low HCB levels during pregnancy.

## 2. Experimental Section

### 2.1. ES Cell Culture

The Wild-type E1 mouse ES cell-line was used for this work. The generation and characterization of E1 ES cells was previously described [15]. ES cells were cultured in Dulbecco’s modified Eagle’s medium (DMEM) supplemented with 15% fetal bovine serum (FBS), nonessential amino acids, 1.0 mM β-mercaptoethanol, and leukemia inhibitory factor (LIF). Medium was changed every two days and the cells were trypsinized when they reached 70% confluency.

### 2.2. GABAergic and Glutamatergic Neuronal Differentiation

To generate GABAergic neurons, undifferentiated ES cells were encapsulated using 1.1% (w/v) alginic acid and 0.1% (v/v) porcine gelatin as previously described [16]. For some experiments, encapsulated ES cells were harvested on day 8 and then transferred to poly-d-lysine (PDL)/laminin-coated plates and cultured in N2 medium (neurobasal medium plus N2 supplement; Invitrogen, Carlsbad, CA, USA) for neuronal selection. After 48 h, the N2 medium was removed and maturation medium (neurobasal medium supplemented with B27; Invitrogen) was added. An additional 5 µM retinoic acid (RA) dose was added at this point for 2 additional days for GABAergic phenotype enrichment. Cells were harvested after four days of culture in maturation medium. To generate glutamatergic neurons, ES cells were grown in low-attachment plates to form embryoid bodies (EBs) and differentiation was carried out as previously described [17].

### 2.3. HCB Treatment and Cell Viability Determination

For these experiments, cells were exposed to either DMSO (0.1%, vehicle) or various HCB doses (0.5, and 1 nM) for 48 h starting on day 1 of the maturation step (*i.e.*, after culture in N2 medium). Determination of cell viability was carried out using the trypan blue exclusion method using a Cellometer (Nexcelom Bioscience, Lawrence, MA, USA). In separate experiments, differentiated cells were harvested for immunofluorescence assays.

### 2.4. Immunofluorescence Analyses

ES cells were fixed in 4% formalin for 15 min, followed by permeabilization for 20 min in 0.1% Triton X-100. Samples were blocked with goat serum and incubated with primary antibodies for 1 h. The primary antibodies used were rabbit anti-GAD 65/67, rabbit anti-vGlut, and guinea pig anti-GABA. Primary antibodies were used at 1:1000 dilutions. The secondary antibodies used were goat anti-rabbit Alexa Fluor 594 and donkey anti-guinea pig Alexa Fluor 488 (Invitrogen). Samples were examined on a Nikon E400 fluorescence microscope.

### 2.5. RT-PCR Analyses

Semiquantitative RT-PCR was performed to examine the mRNA expression levels of different lineage markers in differentiated ES cells at day 8 of encapsulation and after treatment with 0.5 nM HCB or vehicle only. Briefy, total RNA was extracted from the day 8 encapsulated cells using TRIzol reagent (Invitrogen) according to the manufacturer’s protocol. The RNA was used to prepare cDNA and RT-PCR was performed using primers specific for *GAD1* (GABAergic marker), *GAT1* (astrocyte and neuronal marker), *TH* (tyrosine hydroxylase, dopaminergic marker), *Peg1* (or *Mest*, endodermal marker) and *36b4* (ubiquitous housekeeping gene used for loading control). Amplified PCR products were analyzed in 1.2% agarose gels and visualized by staining with ethidium bromide. The sequences of the primers used are: *36b4*, forward 5′-AGAACAACCCAGCTCTGGAGAA-3′, reverse 5′-ACACCCTCCAGAAAGCGAGAGT-3′; *GAD1*, forward 5′-CAGAACCAGAATCATCGGCCAT-3′, reverse 5′-CTGTAGTTGCTTGCGAGATGGT-3′; *GAT1*, forward 5′-TCAGCCACGTACCCCTACAT-3′, reverse 5′-TGCAGCAAACGATGATGGAGT-3′; *TH*, forward 5′-AGGGATGGGAATGCTGTTCTCA-3′, reverse 5′-ACCAGGTGGTGACACTTGTCCAA-3′; *Peg1*, forward, 5′-GCTGCCGCGGTCCACAGTGTC-3′, reverse 5′-GTCACCCTTAGAGATGAGGTGGAC-3′. Amplifications were carried out at 58 °C for 25 cycles.

### 2.6. ROS Assay

Determination of reactive oxygen species was performed using 5-(and-6)-carboxy-2',7'-dichlorodihydrofluorescein diacetate, acetyl ester (CM-H2DCFDA). For this purpose, GABAergic neurons were treated with 0.5 nM HCB or vehicle only for 48 h as described above. After this period of time, neurons were washed three times in sterile PBS for 1 min and then incubated with ROS staining solution (1× PBS, Hoechst 33342 for nuclear staining, and CM-H2DCFDA) for 20 min at room temperature. After incubation, the neurons were washed with sterile PBS once and then viewed using a fluorescence microscope.

### 2.7. NAC Treatment

NAC pre-treatment was used to prevent the formation of reactive oxygen species. After neuronal selection in N2 medium, cells were cultured in maturation medium supplemented with NAC (10 mM). After 1 h, HCB (0 or 0.5 nM) was added to the samples and cells were cultured for an additional 48 h. Next, neurons were washed once with sterile PBS and then assayed for ROS production. In separate experiments, neurons treated with NAC and/or HCB for 48 h were cultured for an additional 48 h in maturation medium only for immunofluorescence analyses.

### 2.8. Neurite Lenght Measurements

Neurite measurements were performed using ImageJ software and were done on five different regions from each independent triplicate experiment. The Images were taken on a fluorescence microscope with a 60× objective. To measure neurite length (in µm), a straight line, calibrated using a scale bar of known dimensions, was drawn from the origen of the neurite in the soma (cell body) to the tip of each neurite. As we obtained neurons with variable number of neurites, on average, three neurites per cell were measured. To determine the total neurite length from each triplicate, the average of the five measurements in both control and HCB-treated groups were taken and results were analyzed by unpaired Student’s *t*-test. Statistical significance was established at *p* < 0.05.

### 2.9. Electrophysiology Recordings

GABAergic neurons were maintained in external solution of the following composition (in mM): NaCl 130, KCl 3, CaCl_2_ 2, MgCl_2_ 0.6, NaHCO_3_ 1, HEPES 10, glucose 5, pH 7.4 adjusted with NaOH. The internal solution contained (in mM): KCl 140, CaCl_2_ 0.1, EGTA 1, MgCl_2_ 2, ATP 2, HEPES 10, pH 7.2 adjusted with Tris. The osmolarity of the solutions were ~300 mOsm/L adjusted with sucrose. Voltage-gated delayed rectifier K^+^ currents were recorded in the tight-seal whole-cell configuration mode at 21–25 °C. High-resolution current recordings were acquired by a computer-based patch-clamp amplifier system (EPC-10, HEKA, Lambrecht, Germany). Patch pipettes had resistances between 3–5 MΩ. Immediately following establishment of the whole-cell configuration, voltage ramps of 50 ms duration spanning the voltage range of −100 to +100 mV were delivered from a holding potential of −80 mV at a rate of 0.5 Hz over a period of 60 s. Data were extracted and analyzed at +50 mV.

## 3. Results

### 3.1. Dose-Response Effect of HCB on Cell Viability

**Figure 1:**
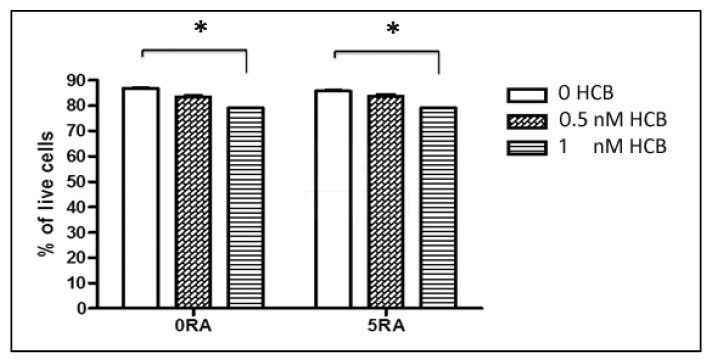
Effect of low dose HCB on ES cell viability. ES cells were encapsulated in hydrogels and grown for 8 days. Cells were treated with various doses of HCB at days 2 and 4 after encapsulation. A set of cells were also treated with 5 µM (5RA) on days 4 and 6 to promote neuronal differentiation. On day 8, cells were harvested, trypsinized, and trypan blue exclusion assay was performed to determine cell viability. HCB at 1 nM, but not at 0.5 nM, significantly reduced cell viability. * *p* < 0.05 by unpaired Student’s *t*-test.

Studies have shown that HCB doses of 10 nM or higher significantly decrease cell viability in a number of mammalian cell types [14,18]. Similarly, previous research in our laboratory (data not shown) indicated that treatment of mouse ES cells for 48 h with HCB doses as low as 2 nM resulted in a high percentage of cell death. In order to identify a sub-toxic concentration of HCB that does not significantly affect ES cell viability, we performed dose-response studies on encapsulated ES cells using 0, 0.5, and 1 nM HCB in the presence or absence of RA (a neuronal inducer). Cells were treated on days 2 and 4 after encapsulation and samples were harvested at day 8 for determination of cell viability. Control cells were treated with vehicle only. As shown in Figure 1, there was no significant decrease in the percentage of cell viability between the control and 0.5 nM HCB treated cells (~85%–88%) in the presence or absence of RA. However, a significant reduction (~78%) in cell viability was observed in cells treated with 1 nM HCB as compared to control cells. Thus, these results indicate that a dose of 0.5 nM HCB does not have an adverse effect on ES cell viability under the tested conditions. As we were interested in examining the effect of sub-toxic HCB doses on ES cell differentiation, we selected the 0.5 nM HCB concentration for further studies.

### 3.2. Expression of Lineage Markers in Encapsulated ES Cells by RT-PCR

**Figure 2:**
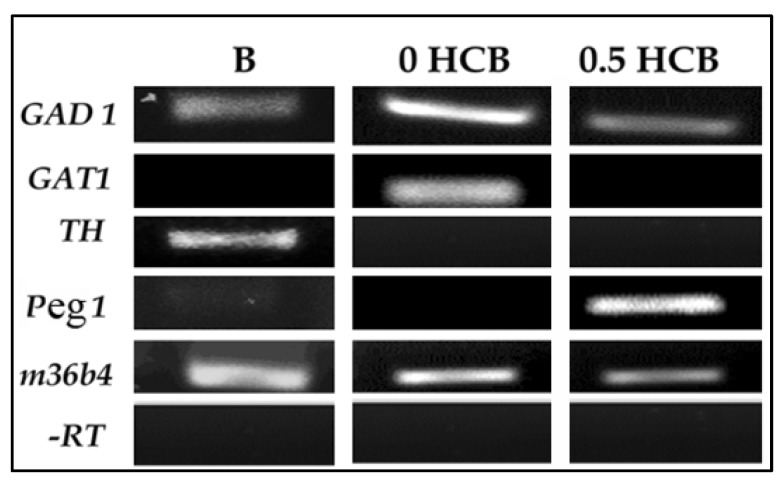
mRNA expression of cell lineage markers in differentiated ES cells treated with HCB. Encapsulated ES cells were treated with 0.5 nM HCB in the presence of RA (5 mM) for 4 days. Total RNA extracted at day 8 after encapsulation was reverse transcribed and used as template for PCR using specific primer pairs for the following markers: *GAD1* (GABAergic neurons), *GAT1* (astrocytes and neurons), *TH* (dopaminergic neurons), *Peg1* (mesodermal marker), and *36B4* (a ubiquitously-expressed gene used as a loading control). –RT, no reverse transcriptase. B, adult mouse brain tissue (positive control). These experiments were performed a minimum of three times starting with cultured cells.

To determine the type of cells obtained from encapsulated cells cultured in the presence of RA plus/minus 0.5 nM HCB, we next employed RT-PCR using primers specific for various cell lineages and neuronal subtypes. We observed that, in the absence of HCB, encapsulated cells produced mostly neuronal subtypes as shown by the expression of *GAD1* (GABAergic marker) and *GAT1* (astrocyte and GABAergic marker) (Figure 2). However, no mRNA expression of the dopaminergic marker *TH* or the mesodermal marker *Peg1* was observed under these conditions. Conversely, treatment with 0.5 nM HCB resulted in a decrease of *GAD1* mRNA expression in day 8 encapsulated cells. HCB treatment also abolished the mRNA expression of *GAT1* but increased *Peg1* mRNA expression. Thus, these results indicate that treatment of ES cells with 0.5 nM HCB during the encapsulation step results in a change of cell fate from neuronal to mesodermal cell lineages.

### 3.3. Low Dose HCB Affects GABAergic Neuronal Maturation

**Figure 3:**
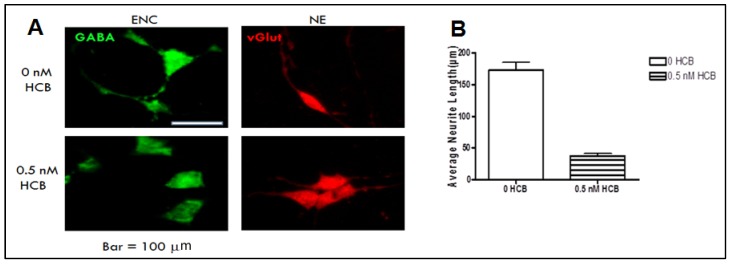
Effect of HCB on neurite outgrowth shown in cells after imunofluorescence. Encapsulated and non-encapsulated cells were treated with 0.5 nM HCB plus RA (5 mM) as described in Materials and Methods, and then harvested at day 8 for further differentiation in N2 medium for 2 days, followed by culture in neuronal maturation medium for 4 days in the presence or absence of 0.5 nM HCB. (**A**) Inhibition of neurite outgrowth by HCB was observed in GABAergic (encapsulated) but not in glutamatergic (non-encapsulated) neurons. GABA = GABAergic marker; vGlut = glutamatergic marker. (**B**) Quantitation of neurite length by ImageJ demonstrates that HCB induces ~3.5-fold reduction in neurite length in GABAergic neurons. *p* < 0.001 by unpaired Student’s *t*-test.

Results from our RT-PCR analyses suggest that HCB interferes with the neuronal differentiation of encapsulated ES cells. In order to investigate whether HCB affects the maturation of cells committed to a neuronal lineage, we next examined the effect of 0.5 nM HCB on the maturation of neuronal precursors *in vitro*. For this purpose, day 8 encapsulated ES cells, previously treated with RA only, were transferred to PDL/laminin-coated plates and grown for 2 days in N2 medium. After this time, the N2 medium was replaced with maturation medium plus or minus HCB and cells were cultured for two days. Next, the medium was replaced by maturation medium without HCB and samples were harvested after two additional days in culture for the determination by immnofluorescence of a GABAergic phenotype in cells obtained from encapsulation *versus* a glutamatergic phenotype in cells obtained from EBs (non-encapsulated). As shown in Figure 3, both encapsulated and non-encapsulated cells differentiated into mature neurons with evident neurite development in the absence of HCB. However, HCB treatment resulted in neurite outgrowth inhibition in GABAergic but not in glutamatergic neurons (panel A). Quantitative analysis of neurite measurements in GABAergic neurons indicate that HCB treatment resulted in 2.3-fold decrease in neurite length as compared to untreated cells. All together, these results show that a low dose of HCB interferes with the normal neurite development of GABAergic, but not glutamatergic, neurons generated from mouse ES cells.

### 3.4. HCB Induces ROS Production in GABAergic Cells

**Figure 4:**
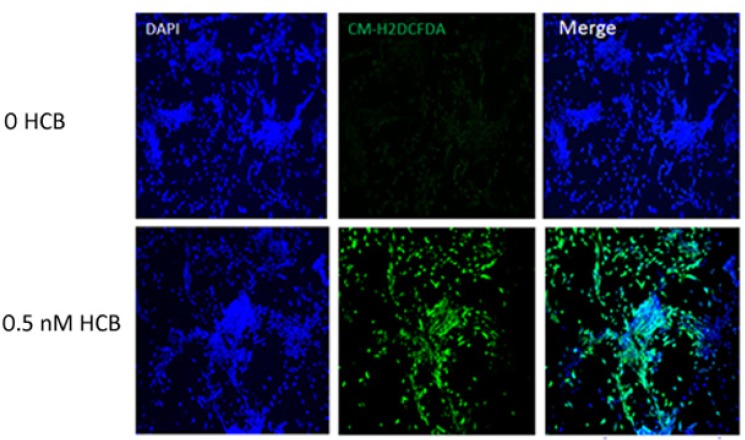
Induction of ROS production by HCB. Encapsulated cells were cultured in maturation medium after selection in N2 medium as described in Materials and Methods. Cells were treated with HCB (0.5 nM) or vehicle only (control) during the first two days of the maturation stage. Next, cells were treated with CM-H2DCFDA to assay for ROS production. ROS production was observed in cells treated with HCB but not in control cells. Cells are shown at the 10× magnification. These experiments were performed at least two times with similar results.

Next, we sought to investigate the mechanism by which HCB interferes with neurite outgrowth in GABAergic neurons. As a number of pollutants and genotoxins have been shown to affect cells by promoting oxidative stress [19,20], we asked whether treatment of GABAergic neurons with 0.5 nM HCB results in the production of reactive oxygen species. For this purpose, neuronal precursors obtained after the N2 stage were treated with 0.5 nM HCB for 48 h and then cells were harvested for ROS assays as described in Materials and Methods. We observed that there was an increase in ROS production in the HCB-treated group but not in the untreated controls as observed in Figure 4. Therefore, these results indicate that HCB induces oxidative stress in GABAergic precursors and suggest that ROS production may repress neurite outgrowth in these cells.

### 3.5. Inhibition of ROS Production Restores Neurite Outgrowth in HCB-Treated Neurons

**Figure 5:**
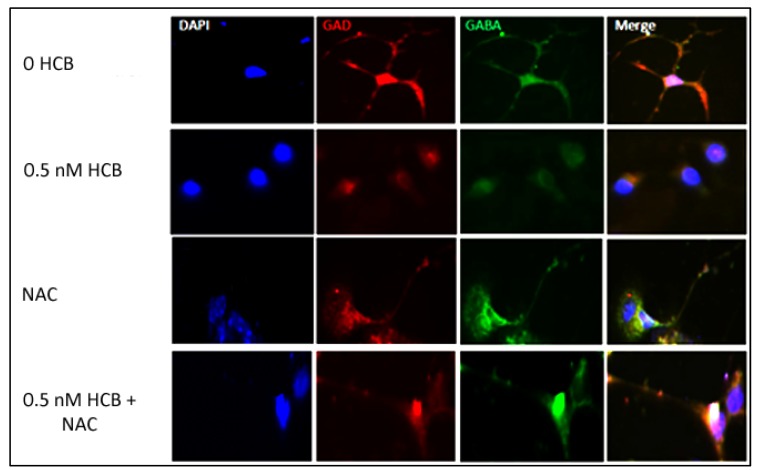
Inhibition of ROS production restores neurite outgrowth. Treatment of cells with NAC restores neurite outgrowth in cells treated with HCB as shown after immunofluorescence. DAPI = nuclear staining; GAD and GABA = GABAergic markers. Magnification = 60×. All panels in Figure 5 are representative results from experiments performed at least three times, starting with cultured cells.

To determine whether ROS production is the main determinant for the HCB-dependent repression of neurite outgrowth in GABAergic neurons, we next pre-treated the neuronal precursors with NAC, a ROS scavenger, for 1 h prior to treatment with 0.5 nM HCB in maturation medium. Cells were harvested 48 h after HCB treatment and then subjected to immunofluorescence analyses for the determination of GABAergic marker expression (GAD1 and GABA). Control cells were treated with NAC or HCB only. As shown in Figure 5, we observed normal dendrite outgrowth in GABAergic neurons cultured in the absence of HCB (control) or in the presence of NAC only. As expected, there was a decrease in average neurite length in neurons treated with 0.5 nM HCB and this effect was abolished by NAC. Expression of GABAergic markers was observed in all cases, which further verifies that HCB at low-doses interferes with neurite outgrowth but not with GABAergic neuronal differentiation. Together, these results suggest that the observed inhibition of neurite outgrowth occurs as a consequence of ROS production induced by HCB in GABAergic neurons.

### 3.6. Electrophysiology Analyses

**Figure 6:**
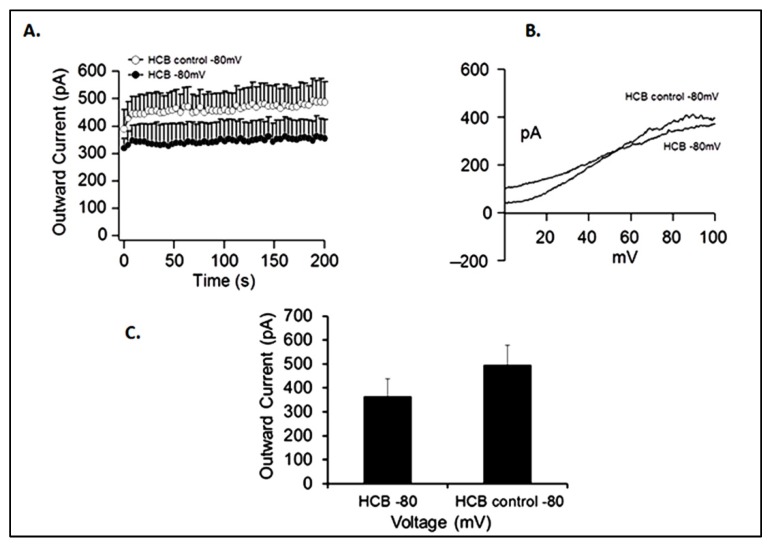
Effect of HCB on voltage-gated delayed rectifier K^+^ currents in neurons obtained after encapsulation. (**A**) Average outward currents recorded from controls and HCB 0.5 nM treated cells extracted at +50 mV from a voltage ramp ranging from −100 mV to +100 mV and −80 mV holding potential (n = 4 cells/group; mean ± SEM). (**B**) Representative current-voltage relationship (I/V) from a cell from each group. (**C**) Average peak currents at 200 s after establishment of whole-cell configuration from panel A (*p* < 0.05). These results indicate that, although HCB affects neurite outgrowth, it does not interfere with the expression of K^+^ channels, which are typically present in mature neurons.

To investigate the effect of HCB on voltage-gated delayed rectifier K^+^ currents, electrophysiology analyses were performed using the whole-patch clamp technique. For this purpose, GABAergic neurons generated in the presence or absence of 0.5 nM HCB were examined by electrophysiology for the presence of functional potassium channels. We observed a large outward K^+^ current in both control and HCB treated groups with a slightly higher outward current in the control group, though this difference was not statistically significant (Figure 6(A) and Figure 6 (C)). Similarly, the observed current-voltage relationship (I/V) values were typical of voltage-gated delayed rectifier K^+^ channels (Figure 6(B)). Taken together, these results demonstrated that although HCB inhibits neurite outgrowth, it does not significantly suppress K^+^ ion channel function, which is indicative of neuronal maturation [21,22,23].

## 4. Conclusions

The implication of HCB exposure with the development of neurological disorders in humans has become evident from cases of Turkish patients exposed to high HCB doses in the 1950s [24,25]. Although the use of HCB and other organochlorine compounds has been restricted in developed and most developing countries since the 1970s [5], HCB is still frequently found at elevated levels in human adipose tissue and breast milk [26] due to its persistent and bioaccumulative characteristics [11]. As many environmental pollutants are active even at extremely low doses [27], we sought to investigate the effect of sub-toxic HCB doses on the maturation of GABAergic *versus* glutamatergic neurons generated from mouse ES cells *in vitro*.

By performing dose-response studies, we found that an HCB concentration of 0.5 nM did not significantly decrease the viability of differentiating ES cells. This low HCB concentration, however, was found by semiquantitative RT-PCR to alter the differentiation fate of encapsulated cells by repressing neural but inducing mesodermal marker gene expression. This effect of HCB may be due to its ability to regulate diverse cell signaling pathways that may be involved in ES cell differentiation along specific lineages. For instance, HCB has been shown to stimulate the insulin pathway [28], which is known to promote the adipose (*i.e*., mesodermal) differentiation of ES cells [29]. Because epidermal growth factor (EGF) signaling is also known to induce mesodermal differentiation in human ES cells, the acquisition of a mesodermal fate by HCB-treated mouse ES cells may also be due to the HCB-dependent stimulation of EGFR trans-activation observed in cultured mammalian cells [30].

Our experiments employing neuronal precursors obtained after culture in N2 medium demonstrated that HCB interferes with neurite outgrowth of GABAergic, but not of glutamatergic neuronal cells. As HCB has been shown to affect c-SRC regulation after binding to the aryl hydrocarbon receptor (AhR) [30], it is possible that the effect of HCB on neurite outgrowth may be a consequence of an alteration in the binding of c-SRC to the neuronal inducer retinoic acid receptor gamma (RARγ) [17], as binding of c-SRC to RARγ has been reported to regulate neurite outgrowth in human neuroblastoma cells [31]. However, the main reason for the observed effect of HCB on GABAergic neurons appears to be the induction of ROS production by this pollutant since the deleterious effect of HCB can be reversed by a ROS savenger. The molecular mechanism by which ROS impairs neurite development is unknown but it may be due to the combined disregulation of multiple signaling pathways known to be affected by oxidative stress (e.g., SRC, ERK, JNK, PKC, and PI3K/AKT) [13,32,33]. The mechanism by which HCB affects neurite outgrowth in GABAergic, but not in glutamatergic neurons is unclear, but is currently been investigated in our laboratory.

In summary, the present work suggest that persistent environmental pollutants such as HCB may interfere with normal GABAergic neuronal function in fetuses exposed through placental transport [25] and may promote the onset of a number of neurological disorders such as autism and ADD.
